# The Contribution of Molecular Biology to Forensic Entomology

**DOI:** 10.3390/insects16070694

**Published:** 2025-07-05

**Authors:** Carmen Scieuzo, Roberta Rinaldi, Federica De Stefano, Aldo Di Fazio, Patrizia Falabella

**Affiliations:** 1 Department of Basic and Applied Sciences, University of Basilicata, Via dell’Ateneo Lucano 10, 85100 Potenza, Italy; carmen.scieuzo@unibas.it (C.S.); roberta.rinaldi@unibas.it (R.R.); federica.destefano@unibas.it (F.D.S.); 2 Spinoff XFlies s.r.l, University of Basilicata, Via dell’Ateneo Lucano 10, 85100 Potenza, Italy; 3 SIC Medicina Legale, (AOR) “San Carlo”, Via Potito Petrone, 85100 Potenza, Italy; aldo.difazio@ospedalesancarlo.it

**Keywords:** forensic entomology, post-mortem interval, cadaveric insects, decomposition, molecular biology

## Abstract

This review presents an in-depth analysis of the synergistic role of molecular biology in advancing forensic entomology. The study discusses how insects associated with decomposing bodies provide critical data for estimating the post-mortem interval (PMI), and how molecular techniques improve species identification and trace analysis. The manuscript examines DNA-based methods such as RAPD, RFLP, and mitochondrial sequencing, along with innovative applications like gene expression profiling and entomotoxicology analysis. Additionally, it presents real case studies illustrating how molecular data from insects can be used not only to estimate PMI but also to identify victims or suspects through human DNA retrieved from insect tissues. These advances confirm the fundamental role of molecular biology in strengthening the reliability and applicability of forensic entomology in legal contexts.

## Simple Summary

This review examines how molecular biology enhances forensic entomology, the study of insects on dead bodies, to determine time since death (post-mortem interval, PMI). Modern molecular approaches, such as DNA barcoding, gene expression profiling, and toxicological analysis, coupled with traditional insect-based research, allow scientists to recover human DNA from insect tissue and identify insect species more accurately, even when they are still in their immature phases. In addition, actual criminal cases where insects were used to identify victims, determine the time of death, or uncover toxic substances are covered in the review. These coordinated strategies show how molecular biology is now crucial to resolving intricate forensic cases.

## 1. Forensic Entomology

Forensic entomology is the study of insects and arthropods associated with decomposing remains to estimate the post-mortem interval (PMI), determine the location and circumstances of death, and provide key forensic evidence [1,2,3]. Insects, due to their species-specific life cycles and predictable succession patterns, offer crucial data for time-of-death estimations [4,5]. Although forensic entomology dates back to the 13th century in China, it gained scientific relevance in the late 19th century in Europe through systematic studies of insect activity on human remains, aiding PMI estimation, body relocation analysis, and toxicological or DNA investigations [6,7,8,9,10].

Decomposition follows standard stages—fresh, chromatic, emphysematous or gaseous (bloated), colliquative (decay and advanced decay), and skeletal (dry) [11,12]—each characterized by physical and biochemical changes that attract specific insect taxa. Decomposing tissues release volatile compounds (e.g., putrescine, cadaverine, indole, and skatole), which produce the typical odour of decay [13].

Insects detect these volatile chemicals by highly sensitive antennae equipped with olfactory receptors and odorant binding proteins, allowing them to locate remains by following odour gradients [14,15,16,17].

Entomotoxicology enables the detection of xenobiotics (e.g., drugs, heavy metals, pesticides) in necrophagous insects, particularly when conventional samples are degraded or unavailable [9,18,19,20,21,22]. Substances such as benzodiazepines, cocaine, or organophosphates can affect larval development, potentially influencing PMI [23,24,25,26,27,28,29,30,31]. Advanced techniques such as GC/MS, HPLC/MS, ELISA, and multi-omics approaches enhance detection accuracy [9,32]. It is important to note that many of the foundational entomotoxicological studies were conducted on animal carcasses (e.g., pigs, rabbits), and metabolic pathways in these models may differ from those in humans, potentially affecting toxicological interpretations. Additionally, human cases often involve the use of drug cocktails, and high inter-individual metabolic variability may significantly alter larval development and drug uptake in insects, resulting in possible inaccuracies in PMI estimation. Forensic entomology is thus not only relevant but essential in modern forensic science, though care must be taken when extrapolating from animal models to human cases [33,34].

Forensic entomology provides key data on PMI, as insect development is highly temperature-dependent [35,36,37,38,39]. The PMI is estimated by comparing the development stage of collected larvae with those reared under known environmental conditions [36,37,38,39,40]. However, once the first adult generation emerges, the ability to distinguish between insect generations diminishes, which in turn compromises the reliability of PMI estimation based on developmental data.

In recent years, attention has also turned to the necrobiome—the network of microbes, invertebrates, and vertebrates that break down carrion [41]. After death, the collapse of the host immune system and environmental exposure lead to microbial succession, which is relatively predictable and can be used to estimate PMI [42]. Microbial data, particularly from soil and insect-associated microbiota, have shown promising results in enhancing forensic resolution, especially when integrated with entomological findings. Fungi also demonstrate distinct successional roles and may modulate insect behaviour or decomposition rates [43]. Moreover, modern techniques such as standardized DNA metabarcoding and machine learning are increasingly used to analyze microbial profiles and improve PMI estimation [44]. Despite challenges such as heterogeneous microbial distribution and limited diversity data, understanding necrobiome dynamics offers a complementary tool for forensic investigation, particularly in indoor or entomologically limited environments.

## 2. Modern Molecular Biology Supporting Forensic Entomology Investigations

Over the past few decades, advances in molecular biology have significantly expanded applications of forensic entomology with equipment that promises higher precision and reliability. DNA-based techniques, such as DNA barcoding, allow for positive insect species identification even from damaged, fragmented, or immature insects [45,46,47] (Table 1). The importance of this lies particularly when morphological identification is ambiguous or when fragments, eggs or pupae are available. Molecular analysis also gives ecological data and helps to comprehend the entomofauna pattern of succession of decomposition [48]. The combination of entomology and molecular methods represents a powerful interdisciplinary strategy, extending the utility of insect evidence to situations such as body relocation, use of narcotics, environmental contamination, and forensic advanced decomposition.

### 2.1. DNA Analysis for the Forensic Identification of Different Dipteran Species

The identification of the insect species during the larval stage can be complex, if not even impossible, through the analysis of morphological characteristics. Molecular biology plays a crucial role by providing an alternative approach for insect identification through DNA analysis [49,50,51,52].

The identification of insect species through DNA relies on specific nucleotide sequences (loci) that constitute the DNA molecule [53,54]. When gene fragments are shorter than 1000 base pairs, they must be amplified using the polymerase chain reaction (PCR) before analysis [35,55]. Once extracted, the insect DNA can be further analyzed through random amplified polymorphic DNA (RAPD) or Restriction Fragment Length Polymorphism (RFLP) analysis [56,57] and also using the Inter Simple Sequence Repeat (ISSR) and Sequence-Characterized Amplified Region (SCAR) markers methods [58]. Moreover, the application of these molecular techniques allows for greater precision in identifying insect species, even in the more challenging stages of their ontogeny, such as the larval stages, and provides essential information for forensic investigations. However, these molecular techniques, next to their advantages, present several challenges and limitations. The accuracy of the analysis can be jeopardised by DNA degradation, particularly in samples exposed to environmental factors like heat, humidity, or microbial activity. False results may arise from contamination during sample collection or laboratory procedures. Furthermore, for many insect species, reference genetic databases are still scarce, which can make definitive identification challenging in certain situations. Importantly, laboratory techniques alone—whether molecular identification or toxicological assays—cannot substitute for expertise in terms of the ecological context, decomposition differences, and insect morphology. For valid casework, trained professionals must integrate lab results with field observations and morphological assessments to accurately interpret forensic entomological evidence [59].

#### 2.1.1. Random Amplified Polymorphic DNA (RAPD)

Currently, insects are the most abundant eukaryotic organisms on Earth, with over 1 million different species described, representing over 80% of all life forms. A major challenge in forensic DNA typing of hexapods is the lack of suitable primers for many species. RAPD (Random Amplified Polymorphic DNA) uses short and arbitrary primers (8–10 bp) that bind randomly across the genome, generating unique patterns across multiple tax [60,61,62]. Unlike species-specific STR primers (Short Tandem Repeat), RAPD primers allow broad applicability and species differentiation. The technique is simple and low cost and requires minimal DNA, making it useful for limited or degraded forensic samples [63,64,65]. RAPD targets the entire genome and has been to differentiate closely related species, including key forensically relevant Diptera. Benecke (1998) [66], the first to use this technique to differentiate insect species of forensic interest, reports some good practices to follow in order to obtain high-quality DNA for amplification and analysis. DNA must be extracted from fresh specimens, both adults and young, collected from decomposing corpses and from dried samples from the same source; when possible, advanced-stage larvae should be used, preferably with empty stomachs, to avoid cross-contamination with foreign DNA [66]. The primers produce a series of bands that can be visualized by agarose gel electrophoresis, and each of the products represents a single genetic locus. RAPD has been effectively used to assess genetic relationships among various dipteran species, including important families like Calliphoridae. Bajpai (2016a) [67] used the RAPD-PCR technique to effectively distinguish two dipterans of the genus Sarcophaga, namely *Sarcophaga albiceps* and *Sarcophaga knabi*. From the 12 primers used, several species-specific bands were obtained, which can be further used for identification purposes [67].

Bajpai (2016b) [68] has also used this technique to establish the genetic relationship between three other species of the Sarcophagidae family, which are important from a forensic perspective: *Sarcophaga ruficornis*, *Sarcophaga argyrostoma*, and *Sarcophaga dux* [68].

##### Identity of the Larvae Found Outside and Inside a Body Bag

In October 1997, a corpse in an advanced state of decomposition was examined to identify traces of insects in order to estimate the PMI [66]. Hundreds of larvae, with an average size of 9 mm, were found both on the body and outside the closed body bag. It is known that larvae can pass through small holes, for example, to find a suitable place for pupation. The larvae outside could have moved through holes coming from inside the bag [64]. Alternatively, a second deposition by another species could have occurred after the body was placed in the bag [66]. Moreover, pupae or empty puparia were found on the floor beneath the corpse that could not be directly associated with a specific body in the morgue, suggesting the possibility that they had fallen from various unknown corpses [58,69]. Since different species of dipterans have varying developmental cycles, estimating the PMI is feasible only by knowing the insect species, which can then be used as a temporal indicator from the time of death [66]. The identification of the species of larvae, especially in the juvenile stage, is complex, which is why a rapid, economical, and reliable DNA analysis test was used [70]. RAPD microspheres were used, known to be a support in situations involving a variety of different species [71]. In this specific case, the larvae from both inside and outside the coffin were examined and subsequently identified as dipterans of the species *Lucilia sericata* [66]. This molecular approach highlights the importance of accurate species identification in forensic entomology, especially when dealing with complex cases where morphological identification may be challenging due to the degradation of specimens or the presence of closely related species [72].

#### 2.1.2. Restriction Fragment Length Polymorphism (RFLP) and Amplified Fragment Length Polymorphism (AFLP)

Restriction Fragment Length Polymorphism (RFLP) is a technique that exploits variations in homologous DNA sequences known as polymorphisms [71,73,74]. The method involves digesting DNA with restriction enzymes and separating the resulting fragments by electrophoresis, allowing genetic variation analysis [71,75]. RFLP is particularly valuable in forensic entomology, where it has been used to identify species involved in criminal investigations through their genetic material [75]. For example, Benkenana (2020) [76] discusses how specific insect species can be tied to the decomposition process and how their identification through molecular methods can aid in estimating the PMI. Similarly, Joseph et al. (2011) [45] provide an overview of how RFLP can be utilized to analyze insect populations, linking genetic profiles to specific forensic cases, which can significantly influence legal outcomes. RFLP had been used for entomological researches: genetic linkage maps in *Bombyx mori* (Linnaeus) [45,76,77,78], Colorado beetle *Leptinotarsa decemlineata* (Say) [79], Colias butterflies [80], phylogenetic studies in mites and ticks [81], and gene flow studies [82] but also in genotyping in forensic entomology such as the study of Ratcliffe et al. (2003) [73] in which the analysis of the internal transcribed spacer regions of ribosomal RNA genes was conducted and the fragments from three restriction digests correctly identified 10 species in the Calliphoridae, Muscidae, and Sarcophagidae families [73] or in the study of Schroeder et al. (2003) [75], in which PCR–RFLP was used for the differentiation and identification of *L. sericata*, *Calliphora vicina,* and *Calliphora vomitoria* on human corpses [75]. Nevertheless, in contrast to PCR-based techniques, this approach requires a substantial amount of high-quality DNA (in µg), the involvement of radioactive material, hazardous chemicals, and comparatively high technical proficiency, which detracts from the appeal of this method [83].

To overcome these limitations, forensic entomologists often integrate RFLP with AFLP (Amplified Fragment Length Polymorphism) [6,84]. AFLP combines restriction digestion with selective amplification and provides high-resolution genotyping across the genome [6,58,85]. Despite being technically demanding, AFLP is repeatable and precise, making it valuable for species identification and population analysis, especially in complex decomposition scenarios [79,86,87].

##### Application of PCR-RFLP Targeting ITS2 for the Forensic Identification of *Chrysomya* Species

A notable case study illustrating the practical application of molecular techniques in forensic entomology is presented by Nelson et al. (2008) [88], who focused on the identification of *Chrysomya* blowfly species using the second ribosomal internal transcribed spacer (ITS2) as a genetic marker. This nuclear marker was employed to address the limitations associated with morphological identification, particularly in the immature stages of development, where closely related species often exhibit few distinguishing features. In their study, Nelson and colleagues amplified and sequenced the ITS2 region from nine Australian *Chrysomya* species, revealing interspecific sequence divergences ranging from 0.23% to 11.82% [88].

A key innovation of this research was the development of a PCR-RFLP (Polymerase Chain Reaction-Restriction Fragment Length Polymorphism) protocol using five restriction enzymes, DraI, BsaXI, BciVI, AseI, and HinfI, which produced species-specific restriction profiles for most of the species examined. For example, digestion with DraI successfully differentiated *Chrysomya incisuralis* and *Crysomya rufifacies* (both presenting three restriction sites) from *Chrysomya megacephala* and *Chrysomya flavifrons* (both presenting no restriction sites), while HinfI enabled the distinction between *Chrysomya latifrons* and *Chrysomya semimetallica* (one restriction site detected). Nevertheless, the protocol encountered limitations when attempting to differentiate two closely related species pairs, *C. latifrons* + *C. semimetallica* and *C. incisuralis* + *C. Rufifacies*, due to minimal sequence divergence. However, amplification of the entire ITS region, which revealed size differences (1036 bp versus 1150 bp), successfully resolved the latter pair [88].

This case also demonstrates strong coherence with mitochondrial DNA (mtDNA) approaches. While mitochondrial markers such as COI and COII have been widely used to differentiate broader taxonomic groups and geographic variants, as seen in the work of Park et al. (2009) [89] on *Lucilia* species, the study by Nelson et al. (2008) [88] highlights the complementary role of nuclear DNA markers like ITS2. Nuclear markers can provide crucial resolution among closely related species whose mitochondrial genomes are highly conserved. This underscores the value of adopting a multi-locus strategy, integrating ITS2, mitochondrial COI sequences, and morphological traits, particularly when addressing challenges such as intragenomic variation, as observed in *C. flavifrons*, and the occurrence of overlapping haplotypes [88,89].

From a forensic perspective, the PCR-RFLP protocol developed by Nelson et al. (2008) [88] offers significant advantages. It enabled the identification of species within approximately six hours, offering a rapid and cost-effective alternative to sequencing methods. Such efficiency is critical in forensic investigations, particularly in the estimation of the PMI, where timely analysis of entomological evidence is essential [88].

#### 2.1.3. Analysis of Mitochondrial DNA

The mtDNA of insects, inherited maternally and comprising ~16,000 base pairs, contains genes used in forensic identification, including those coding for cytochrome c oxidase subunits I and II (COI and COII) [90,91]. COI, in particular, is widely used due to its mix of conserved and variable regions, making it suitable for species-level resolution [92,93]. Mitochondrial markers like COI have been increasingly applied to distinguish morphologically similar immature flies [89,94]. Park et al. (2009) [89] sequenced the full-length COI gene of Korean Luciliinae flies, including species like *Lucilia caesar* and *Lucilia illustris*, to address inconsistencies in morphological identification. Their study revealed that COI sequences could distinguish these species but highlighted challenges such as overlapping haplotypes in closely related taxa and geographic variations, emphasizing the need for additional nuclear markers [89].

Boehme et al. (2011) [94] demonstrated the utility of a 658 bp COI “barcode” region for identifying forensically important Diptera in Germany, including *Lucilia* species. Their results showed that all analyzed species formed distinct monophyletic clades, even closely related sister species like *L. caesar* and *L. illustris*, which had previously been difficult to differentiate. The study also noted significant intraspecific variation in some species, underscoring the importance of region-specific genetic databases [94].

In insects, the non-coding region of the mtDNA structure is called the control region (CR), known for its greater variability among species. This region is also known as the “A-T region”, as it is rich in adenine and thymine nucleotides and controls mtDNA replication and RNA transcription [95]. To describe the sequence of base pairs, so that an individual “signature” or haplotype can be specified for a particular species, a nucleotide position numbering system is used.

The mutation rate of mtDNA is such that it can distinguish between genetically close insect species [53,95]. For example, it was successfully used by Avise et al. (1987) to distinguish between *Phormia regina*, *L. sericata*, and *L. illustris* [95].

Once extracted, the sequences of the protein-coding regions of mtDNA, such as those coding for the COI and COII subunits, are compared with the sequences of known species in the GenBank database using computer software (GenBank Release 122, February 2001).

Also, the study of Preativatanyou et al. (2010) [96], through the application of partial mitochondrial COI and COII sequences, led to the differentiation of three common forensically important blowfly species in Thailand, *C. megacephala*, *C. rufifacies,* and *L. cuprina,* and this underlined the importance of using a broad enough genetic database of all relevant species as an essential key for accurate species identification by the phylogenetic analysis of the COI sequence [96].

Similarly, studies such as that of Wallman and Donnellan (2001) [97] have demonstrated the utility of partial COI and COII sequencing in distinguishing among blowfly species in southeastern Australia, including *Calliphora*, *Chrysomya*, and *Onesia* species. Their results showed that sequence variability within these genes allowed reliable classification into species groups, supporting established taxonomy. However, resolution was limited in cases of closely related taxa, where genetic divergence was low (e.g., *Calliphora augur* vs. *Callyphora dubia*), indicating a need for additional genetic markers [97].

In line with these findings, Wells and Sperling (2001) [98] also highlighted the value of COI and COII markers for identifying North American blowfly species. Their work confirmed that interspecific variation greatly exceeded intraspecific differences, and their phylogenetic analyses supported species-level separations. These results affirm that mtDNA is a powerful tool in forensic entomology, particularly when supported by comprehensive and curated genetic databases [98].

##### Differentiation of *Hemilucilia segmentaria* and *Hemilucilia semidiaphana*

The identification of certain insect species, especially in their immature stages, can be complicated. Species of the same genus, such as *Hemilucilia segmentaria* and *Hemilucilia semidiaphana*, dipterans belonging to the family Calliphoridae, are morphologically and behaviourally very similar, but they differ in their growth and maturation rates [99].

For the identification of these two insects, the sequences obtained through the amplification of two specific regions of mtDNA, the COI region and the CR region, which plays a regulatory role in replication and transcription processes, were analyzed using the PCR technique [99]. The amplicons resulting from the amplification of the COI gene region exhibit a constant size of approximately 880 bases in both species, indicating a conservation of length within each species [99].

The amplicons of the CR region show different sizes for the two species: approximately 560 bases for *H. segmentaria* and approximately 450 bases for *H. semidiaphana*. This size difference provides an initial distinguishing marker between the two species and demonstrates the conservation of this characteristic within each species [99].

Following amplification, the specific mtDNA sequences (COI and CR) were treated with four different restriction endonucleases (Dra I, Eco RV, Ssp I, and Taq I) to search for restriction sites that could uniquely confirm the difference between the two *Hemilucilia* species [99].

The digestion with DraI and Ssp I of the CR produced restriction patterns that were sufficient to distinguish the two species. Indeed, the use of these endonucleases generated species-specific fragments, thus allowing for unequivocal differentiation [99]. The digestion with Ssp I of the COI region also produced restriction fragments sufficient to distinguish the two different species. This indicates that, in the COI region, there are detectable sequence differences between the two species that can be highlighted through digestion with Ssp I [99].

At the end, the amplicons of the COI and CR genes of both *Hemilucilia* species were treated with the endonucleases EcoRV and TaqI. The application of the EcoRV endonuclease on the COI region revealed polymorphic patterns, indicating variations in the restriction sites between the COI sequences of the two species. The presence of polymorphic variants suggests genetic diversity within this region [99].

The CR region, subjected to analysis with the EcoRV endonuclease, did not show any restriction sites. This result indicates that, with this specific endonuclease, the control region is similar in both species of *Hemilucilia*.

Although the COI region presents two restriction sites for the TaqI endonuclease, both *Hemilucilia* species showed the same monomorphic pattern. This means that there has been no variation in the TaqI restriction sites, leading to a lack of discrimination between the two species using this specific endonuclease in the COI region [99]. In the CR of both species, no restriction sites for the TaqI endonuclease were identified. These results, combined with previous analyses, indicate that TaqI is not useful for distinguishing species, neither in the COI region nor in the CR [99].

##### A Real Case: Molecular Identification of Scuttle Flies (Diptera: Phoridae)

A notable contribution to the forensic application of molecular techniques is presented by Boehme et al. (2010) [100], who explored the use of DNA barcoding based on the mitochondrial COI gene to identify Phoridae flies. Their study underscores the efficacy of the COI barcode in the identification of six forensically relevant Phoridae species—*Megaselia scalaris*, *Megaselia giraudii*, *Megaselia abdita*, *Megaselia rufipes*, *Conicera tibialis*, and *Puliciphora borinquenensis*—a group often overlooked in forensic investigations due to difficulties in morphological identification. This work is especially valuable in cases where blowflies are absent or access to remains is restricted [100].

The methodology involved extracting genomic DNA from adult specimens, amplifying a 658-base pair fragment of the COI gene using universal primers (LCO1490 and HCO2198), and subsequently sequencing and aligning the fragments. Analysis of interspecific and intraspecific variation was performed through phylogenetic tree construction (e.g., neighbor-joining methods) and the calculation of pairwise divergence metrics. The results revealed high interspecific nucleotide divergence, ranging from 7.9% to 18.6%, which enabled clear differentiation between species. For instance, *M. giraudii* and *M. rufipes* exhibited 7.9% divergence, while *P. borinquenensis* and *M. rufipes* displayed a divergence of 18.6%. In contrast, intraspecific variation was minimal (generally less than 1%), with the exception of *M. rufipes*, where minor haplotype differences (one to two base pairs) were observed [101].

The forensic utility of the COI barcode was clearly demonstrated, as it reliably distinguished species that are often morphologically indistinguishable, such as within the *Megaselia genus*. The practical implications of this approach were illustrated through several case applications. In one case, the presence of *M. abdita* on a mummified corpse indicated a death occurring during colder months when blowfly activity would have been minimal, aligning with investigative timelines. In another case, the infestation of a concealed newborn’s body by *C. tibialis* and *M. scalaris* suggested a PMI of approximately four months, a finding corroborated by developmental data. Furthermore, in a third case, the co-occurrence of *C. vicina* (with a development time of five days) and *M. scalaris* pupae (developing over 10 to 11 days at 20 °C) helped forensic investigators reconcile the PMI with suspect activity timelines [100].

In conclusion, the study by Boehme et al. (2010) [100] underscores the efficacy of the COI barcode in the identification of Phoridae species: by enabling precise species identification, this molecular tool enhances the accuracy of PMI estimations and supports forensic investigations. The authors also advocate for the expansion of reference databases to maximize the forensic utility of scuttle flies. Integrating this work within the broader context of forensic entomology, it parallels findings in blowfly studies (e.g., *Hemilucilia* spp.), which also demonstrate how mitochondrial markers like COI can overcome limitations inherent to morphological identification. Nevertheless, the study also highlights the necessity of incorporating complementary nuclear markers in instances of low interspecific divergence, as observed in other Diptera research, such as *Lucilia* species. Overall, the combination of COI barcoding and developmental data firmly positions Phoridae as valuable forensic indicators, particularly in cases involving concealed or buried remains [100].

#### 2.1.4. Inter Simple Sequence Repeat (ISSR) and the Sequence-Characterized Amplified Region (SCAR)

Inter-simple sequence repeats (ISSRs) are DNA fragments, typically 100–3000 bp long, found between adjacent microsatellite regions that are oriented in opposite directions [58]. Variations in these inter-microsatellite areas are used for genotyping by the ISSR-PCR method. With a few specific nucleotides serving as anchors in the adjacent non-repetitive areas, typically spanning 16–18 base pairs, the primers employed in this technique are based on microsatellite core sequences [35]. The fact that ISSRs do not require previous sequence information for constructing primers is one of their main advantages; also, comparing electrophoretic profiles to reference samples from the same species is the basis for ISSR identification [58,101]. This method needs a thorough database of animals frequently found in the same geographic area as corpses in order to guarantee high dependability. On the other hand, Sequence-Characterized Amplified Regions (SCARs) are genomic fragments that have been amplified from certain loci using specific primers. Regardless of the reagents or equipment employed, SCARs ensure superior repeatability across laboratories since they are less susceptible to changes in reaction circumstances than ISSR markers [102]. In applied forensic situations, SCARs are therefore seen as a more reliable and useful tool for species identification [103,104,105].

For example, this method was applied to analyze DNA polymorphism among five forensic fly species in China: *Phaenicia sericata*, *Aldrichina grahami*, *C. megacephala*, *Parasarcophaga crassipalpis*, and *Musca domestica*. Using nine ISSR primers, researchers identified 95 polymorphic bands, which proved effective for species differentiation. Additionally, they converted ISSR fragments specific to each species into sequence-characterized amplified region (SCAR) markers, enabling the molecular identification of these flies [106].

### 2.2. The Case of Phormia regina

Microsatellite markers have emerged as a powerful tool in forensic entomology, with particular significance for species such as *P. regina*, a blow fly commonly associated with the early stages of decomposition. In their 2014 study, Farncombe et al. (2014) [107] developed novel microsatellite markers for *P. regina* using next-generation sequencing (NGS). After identifying and testing 84 candidate loci, they selected 14 polymorphic markers that were reliably amplifiable for further analysis. These markers exhibited substantial genetic variability, with observed heterozygosity ranging from 0.385 to 0.909 and between 4 and 26 alleles per locus, demonstrating their potential for use in population genetic studies. The authors emphasized the importance of these markers in forensic investigations, including their ability to estimate PMI or detect corpse relocation through the comparison of genotypic profiles from larval populations across different geographic regions. This research marks a significant advancement in the molecular application of *P. regina* in forensic entomology, laying the groundwork for future studies in this field [107]. However, despite their promise, microsatellite markers also present challenges and limitations. The development of species-specific microsatellites is time-consuming and resource-intensive, requiring comprehensive genomic information. Microsatellite analysis can be affected by issues such as null alleles and allele dropout, which complicate genotyping and data interpretation. Furthermore, these markers may show limited transferability across populations due to regional genetic differentiation. The need for high-quality DNA samples is critical, and degraded or contaminated samples may yield unreliable results. Finally, the cost and technical expertise required for microsatellite genotyping may limit its accessibility in some forensic laboratories, particularly in resource-limited settings.

### 2.3. Gene Expression in Forensic Entomology

The accurate estimation of insect developmental age is critical in forensic entomology, as it directly influences the reliability of PMI calculations. Traditional methods rely on morphological and morphometric analyses, which are often limited in precision, particularly for stages with minimal external changes, such as eggs and pupae. Recent advances in molecular biology have introduced gene expression profiling as a robust alternative, enabling the identification of age-specific transcriptional signatures that correlate with developmental progression. Studies have demonstrated that key developmental genes exhibit temporally regulated expression patterns, allowing for fine-scale age estimation. For example, Tarone et al. (2007) investigated the expression of *bicoid (bcd)*, *slalom (sll)*, and *chitin synthase (cs)* in *L. sericata* eggs and found that transcript levels followed predictable trends: *bcd* and *sll* were highly expressed in early embryogenesis but declined over time, whereas *cs* was absent in freshly laid eggs but increased significantly as cuticle formation commenced [108]. By integrating these expression profiles into generalized additive models, the researchers achieved age predictions within a 2 h margin of error, a level of precision unattainable through traditional visual inspection [108]. Similarly, Zehner et al. (2009) [109] employed differential-display reverse transcription PCR (ddRT-PCR) and quantitative real-time PCR (qPCR) to identify nine differentially expressed genes (DEGs) in *C. vicina* pupae, revealing distinct transcriptional patterns at early (24 h), mid (120 h), and late (216 h) pupal stages. These findings underscore the potential of gene expression as a molecular chronometer for insect development [109]. However, this approach is not without its limitations. Gene expression can vary significantly depending on external conditions, making it essential to control for environmental influences when interpreting results. Moreover, inter-individual biological variation and the temporal overlap in expression levels between adjacent developmental stages may reduce the resolution of age estimates. These factors highlight the need for standardized protocols (beyond the standardized procedures for insect collection and preservation, and rearing of specimens to adulthood) and validation across multiple populations before gene expression profiling can be fully integrated into forensic casework [59].

#### Enhancing PMI Estimation in Forensic Investigations

A practical demonstration of this approach was documented by Zehner et al. (2009), where gene expression analysis was applied to pupae recovered from a forensic case [109]. Traditional methods would have required rearing the pupae to adulthood to determine their age, a time-consuming process with potential complications due to environmental variability. Instead, qPCR was used to quantify DEGs, such as those showing upregulation during mid-metamorphosis (DEG6, DEG8) or downregulation in late pupation (DEG4, DEG5). By comparing these expression profiles to a calibrated developmental timeline, investigators could assign an age range to the pupae with high confidence. This method not only accelerated the PMI estimation process but also improved its accuracy, particularly in cases where rearing was impractical [110].

The integration of gene expression data into forensic entomology represents a significant advancement, offering a reproducible, quantifiable, and Daubert-compliant approach to developmental age estimation. Future research should focus on expanding the catalogue of age-informative genes and optimizing high-throughput techniques, such as RNA sequencing, to further refine PMI predictions in medicolegal investigations [110].

### 2.4. Detection of Human DNA in Insects of Forensic Interest

Insects have historically provided crucial information for determining the PMI in criminal investigations. However, the field of entomology has evolved, recognizing the role of insects as vectors of human and animal DNA through the consumption of biological material [111]. Foreign DNA can be extracted and analyzed from all stages of the insect life cycle and even from their feces [111,112]. Until today, DNA recovered from insects has been successfully used for forensic investigations. Moreover, the potential use of DNA extends to the possibility of identifying perpetrators, confirming the food source of insects to assess the PMI, identifying crime scenes, and establishing connections between individuals and locations [111]. It should be emphasized, however, that insects that have consumed biological material may transfer DNA as they move, creating potential risks of contamination of forensic evidence, complicating investigations, and potentially leading to errors in the incrimination or exclusion of individuals [111]. Therefore, it is of fundamental importance to raise awareness not only about the utility of insects as vectors of DNA of forensic interest but also about the potential risks of contamination in this context [113]. In addition, several methodological limitations should be considered. The quantity and quality of retrievable human DNA from insects can be highly variable and often degraded, depending on the digestion time and the physiological state of the specimen. Differentiating between endogenous insect DNA and exogenous human DNA requires precise analytical strategies, and the presence of inhibitors in insect tissues may interfere with downstream molecular analyses. Moreover, while mtDNA is commonly used due to its higher copy number, its lower discriminatory power compared to nuclear DNA can restrict individual identification in complex forensic scenarios. These aspects highlight the necessity for rigorous validation and careful interpretation when incorporating insect-derived DNA into evidentiary frameworks.

A specific example of the application of DNA analysis is reported by Wells and colleagues (2001) [114], who demonstrated that it is possible to recover DNA from corpses through insect larvae using mtDNA. In their research, they used a living donor from whom the liver had been removed for transplant purposes. The fly larvae were fed on the liver removed from the donor, and the intestinal contents of these larvae were subsequently analyzed. The control sample is represented by the DNA from the blood sample taken from the human patient who underwent the transplant. It was found that the DNA from both sources (larvae fed on the patient’s liver and the patient’s blood) exhibited the same characteristics [114].

The same authors applied this technique to confirm the presence of a corpse at a crime scene. In 1989, a collection of larvae, later identified as *Chrysomya. albiceps*, was found on the floor of a cellar in a farmhouse in southern Italy. The police, acting on a tip-off, conducted a search of the farmhouse but found no body [24,115,116]. This happened because, in the meantime, the perpetrators had moved the body. Police officers skilled in entomology collected the larvae from the basement floor in an attempt to link the location to the victim’s previous presence. The DNA content of the dipteran larvae was analyzed alongside the material found from the missing body. The results confirmed that both DNA samples came from the same sources [113,114,115,116].

A relevant real case is that of Florida, in which maggots on a decomposing body helped solve a murder case. Researchers were able to extract human DNA from the larvae that had fed on the body and ultimately helped identify the victim and supply key evidence [117]. Bini et al. (2021) [117] developed a DNA-based method to distinguish fly artifacts (such as regurgitation or defecation spots) from human bloodstains at crime scenes. Their study demonstrated that human DNA could be successfully extracted and profiled from fly artifacts, even when mixed with insect DNA, using STR analysis. This approach is crucial for avoiding misinterpretation of biological evidence, particularly in cases where fly activity could contaminate or alter bloodstain patterns [117].

Similarly, the study by Kester et al. (2010) revealed that human nuclear DNA was found in 89% of environmental samples collected via insects, highlighting their ability to capture and preserve DNA in a wide range of forensic contexts [118]. Kester et al. (2010) [118] investigated the recovery of human DNA from insects exposed to environmental samples, including flies and beetles. Their findings showed that human DNA could persist in the digestive tracts of insects for up to 14 days post-feeding, with successful STR profiling in most cases. This study underscored the potential of entomological evidence not only for PMI estimation but also for linking suspects or victims to crime scenes through trace DNA transfer [118].

This is echoed by the research of Durdle, which positions insects as having double value: they are used as indicators of PMI and as carriers that are capable of transferring human biological material, thus linking victims, or even suspects, to crime scenes [110]. The value of insect gut contents to identify attackers offers a valuable addition to the crime scene investigator’s arsenal, especially where traditional biological evidence is degraded or not present.

The study by Park et al. (2013) [119] demonstrated that the bicoid gene—a key developmental regulator in insects—exhibits sufficient interspecies variation to distinguish forensically important blowflies (Calliphoridae), even in suboptimal conditions. By sequencing a 658-bp fragment of bicoid, the researchers achieved accurate species identification, which is essential for improving PMI estimates, as developmental rates are species-specific; linking insect evidence to geographic locations, since blowfly species have distinct distributions; and resolving mixed samples, where multiple species may coexist. This genetic approach is particularly valuable in cases of advanced decomposition, where morphological features are obscured. Moreover, it complements other molecular techniques like COI barcoding, offering a multi-locus strategy for robust forensic conclusions [119].

In blood-feeding insects like mosquitoes or triatomines, the detection of human or mammalian DNA by PCR analysis of blood meals has been demonstrated to retrieve DNA even hours or days after feeding, which is especially useful in cases of abduction or in the identification of victims in enclosed areas [120,121]. Studies have shown that mtDNA and nuclear microsatellites can be successfully amplified from blood-fed insects, enabling not only species identification but also individual genetic profiling. This approach proves particularly effective when victims have been held in isolated areas, as insects may be the only available carriers of trace human DNA. For instance, in forensic casework, triatomine bugs collected from crime scenes have provided crucial DNA evidence linking suspects to specific locations [120]. Similarly, *Aedes aegypti* mosquitoes have been used to recover human DNA up to 36 h post-feeding, demonstrating the stability of genetic material in the insect’s digestive tract [121]. Additionally, protein mass spectrometry has been employed to identify blood meal sources, complementing DNA-based methods and enhancing the reliability of forensic analyses [122].

Moreover, the analysis of insect gut contents has been validated in a number of studies. For example, Linville et al. (2004) succeeded in recovering human DNA from the guts of maggots, and the same was conducted through similar research to prove the recovery of mtDNA even from the subsequent stages of decomposition [123,124]. The aforementioned demonstrate how samples obtained from insects not only aid PMI estimates but also enhance the value of forensic genetic analysis through linking biological evidence with victims and suspects [125].

Findings by Campobasso et al. (2005) [126] indicate that host DNA can be successfully analyzed in maggots that are fully developed and actively feeding on a corpse. However, this becomes less feasible in postfeeding or starved maggots with empty crops (≤1 mm), especially after 24–48 h without food. Since crop contents diminish rapidly within this timeframe, the immediate preservation of maggots at the crime scene is essential. Examining crop morphology prior to gut-content analysis may aid in both host DNA genotyping and larval age estimation. It is important to consider species-specific differences in crop emptying rates; for instance, *L. sericata* clears its crop quickly post-feeding and *C. rufifacies* more gradually. Targeting the crop for DNA extraction is recommended over using the entire maggot, as this approach preserves key external features used in species identification and captures less digested material. Additionally, host mtDNA has been successfully retrieved from beetle larvae found on decomposed human remains, indicating that mtDNA can persist and be recovered even in late stages of decomposition and from later colonizing insects. Regarding DNA degradation during digestion, there is no significant enzymatic breakdown of host DNA in the crop over time. This is because the crop primarily serves as a food reservoir and does not receive proteolytic enzymes, which are absent in this part of the foregut. However, digestive enzymes present in maggot saliva for preoral digestion may enter the crop along with ingested food [126,127].

In short, the incorporation of DNA analysis of insect samples in forensic protocols is a groundbreaking action in legal medicine. The ability of insects to store human DNA from the surroundings or diet gives an alternative and complementary means of evidence collection, particularly in contaminated or degraded crime scenes. With growth in forensic entomology, the interaction between entomological data and molecular biology will be increasingly critical in solving crimes and administering justice.

#### 2.4.1. Identification of the Aggressor

Human DNA recovered from insects could be useful in identifying an assailant. Semen, for example, is attractive to dipterans, placing this material in a prominent position as a possible source of valuable genetic material in the context of investigations related to cases of sexual assault. It is important to note that human sperm, once present on a corpse, will undergo a degradation process during the body’s decomposition [128]. The importance of this dynamic is particularly evident in cases where a sexual assault occurred before the victim’s death; in such circumstances, the risk of losing crucial evidence increases significantly if the body begins to decompose before being discovered. However, by consuming the seminal fluid, insects could act as “guardians” of the DNA contained within the sperm. Although it has been shown that dipterans exhibit a strong attraction to sperm as a food source, a high mortality rate has also been reported in *L. cuprina* specimens, and it has been observed that some dipterans of this species appear to suffer paralysis in some legs after feeding on sperm samples [111]. It is possible that the impact is due to the high DNA content in the sperm [129], as guanylic acid, a component of DNA, is known to be toxic to many organisms [130].

#### 2.4.2. Identification of the Victim

In a 2013 report, it is described how DNA genotyping analysis of the stomach contents of dipteran larvae was used in a criminal investigation to identify a charred human corpse [111]. Because of the condition of the corpse, only a small portion of the liver was suitable for DNA analysis, but efforts to obtain a genetic profile were unsuccessful. Three larvae belonging to the families Calliphoridae and Sarcophagidae recovered from the body and that were preserved were subjected to DNA analysis, and it was possible to obtain a partial profile with 12 out of 15 loci, including amelogenin (a gene coding for tooth enamel). Although a reference profile of the alleged victim was not available, the profile was matched to that of the father with a calculated paternity probability of 99.685%.

### 2.5. The Use of Allozymes to Identify Insect Species

DNA is not the only molecular method used to characterize insects. Allozymes, different forms of the same enzyme coded by different alleles at the same gene locus, for example, are enzymes that, due to a genetic mutation, can show variations within individual species. The differences in enzymes can be investigated using electrophoretic techniques such as isoelectric focusing (an electrophoretic protein separation technique based on their difference in isoelectric point) and protein isolation in bands on polyacrylamide gel [35].

Allozymes have been used in some studies to identify a range of cadaveric dipterans [131]. Four species of diptera were studied: *C. dubia*, *Calliphora stygia*, *Calliphora hilli hilli*, and *C. vicina*. Using 42 allozymes, Wallman and Adams were able to show a clear distinction between these species. Through the electrophoretic analysis of allozymes, it has been demonstrated that the larvae of some species of Diptera exhibit genetic profiles that are similar or identical to those found in the adults of the same species [131]. This consistency in genetic profiles between larval and adult stages has allowed for the distinction and identification of the larvae of these dipteran species by comparing them with the known adults of the same species [131]. Nonetheless, the allozyme technique is not without its limitations. Compared to DNA-based techniques, allozymes are generally less discriminatory, particularly at distinguishing between closely related taxa. The breakdown of proteins poses a major limitation because the method requires fairly fresh or stored specimens in order to maintain enzyme integrity. The electrophoretic banding may further pose difficulties in interpretation owing to overlapping bands or variance in expression of the enzyme, thereby sacrificing reproducibility. The scope of allozyme analysis is subsequently restricted by the need for polymorphic enzymes within the target species. Such considerations can render allozyme methods unrealistic, especially in forensic science, where sample quality and quantity might be less than optimum.

## 3. Conclusions

Forensic entomology has grown considerably with the incorporation of molecular biology, thus making the science more accurate, reliable, and useful. Molecular techniques such as RAPD, RFLP, mtDNA analysis, gene expression profiling, and omics-based techniques now enable us to identify species properly—even at the immature or degraded stages—and also for the proper estimation of the PMI. Furthermore, these tools have enhanced the use of insects in forensics so that human DNA may be recovered to identify victims or suspects and important toxicological and environmental data from the body obtained by entomotoxicology and necrobiome analysis. The real forensic cases presented here in this review demonstrate how molecular methods can help explain complex situations like body movement, advanced decomposition, or DNA degradation in traditional samples. These cross-disciplinary methods show that insects are not only temporal markers but also biological carriers of forensic information. In the years to come, additional advances in standardized techniques, regional-level genetic databases, and high-throughput analytical instruments will be essential to move molecular entomology into broader acceptance in forensic applications. Finally, the incorporation of molecular biology strengthens the scientific integrity and admissibility in court of forensic entomology, which makes it an essential tool in modern forensic science.

## Figures and Tables

**Table 1:** Comparative summary of molecular biology techniques in forensic entomology.

Method	Application Scenarios	Advantages	Limitations
RAPD	Species differentiation in larvae; rapid screening in forensic casework.	Low cost, no sequence data required, fast; low DNA quantity needed.	Low reproducibility; random binding; sensitive to contamination.
RFLP	Identification of Diptera species using ribosomal regions; phylogenetics; identification in forensic insects.	High specificity; reliable for known species with reference digestion profiles.	Requires high-quality DNA; time-consuming; radioactive/hazardous materials possible use.
AFLP	Genetic diversity and species identification; used in geographically localized entomological studies.	Can detect many loci genome-wide; no prior sequence data required; reproducible.	Technically demanding; time-consuming; needs extensive optimization.
mtDNA Analysis (COI/COII)	Distinguishing morphologically similar or immature insects; useful for degraded samples.	Maternal inheritance, conserved markers; effective even in poor DNA samples; high copy number; well-documented loci.	Low interspecies divergence in some groups; requires regional databases.
ISSR/SCAR	Species identification without prior sequence knowledge; conversion to SCAR enhances reproducibility.	No need for sequence data; SCARs more robust across labs.	Requires reference electrophoretic database; few studies on forensic species.
Allozymes	Larvae-adult species matching; older technique for species-level separation.	Simple setup; effective and inexpensive if enzymes are polymorphic; direct comparison larvae/adult.	Low resolution; fresh samples required; overlapping band issues.
Microsatellites	PMI estimation, geographic origin tracing, corpse relocation studies.	Highly polymorphic; informative markers; strong in population studies.	Laborious development; may show null alleles; costly genotyping.
Gene Expression Profiling	Estimating precise age of eggs or pupae to refine PMI calculations.	High temporal resolution (±2 h); non-invasive; avoids rearing to adulthood.	Sensitive to environment/individual variation; requires standardization.
Detection of Human DNA in Insects	Victim/suspect identification via DNA from larvae, pupae, or feces.	DNA can be recovered long after ingestion; extends utility in degraded scenes.	Risk of contamination; requires clean separation of human/insect DNA; low yield.
Necrobiome Metabarcoding	PMI estimation using microbial succession; body relocation inference.	High resolution; complementary to insect data; applicable indoors.	Microbial distribution varies; needs bioinformatics pipelines and large databases.
Fly Artifact DNA Analysis	Discrimination of human blood vs. fly artifacts; crime scene reconstruction.	Avoids false positive bloodstain analysis; enhances trace evidence validity.	DNA mixing from insect/human; small sample; needs high-fidelity profiling.
Bicoid Gene Analysis	Species identification when COI insufficient, especially in advanced decomposition.	Nuclear marker complements mtDNA; discriminates similar species.	Less established; requires sequencing; not yet standardized in casework.

## Data Availability

No new data were created or analyzed in this study. Data sharing is not applicable to this article.
